# RNA Interference Therapy for Chronic Hepatitis B Predicts the Importance of Addressing Viral Integration When Developing Novel Cure Strategies

**DOI:** 10.3390/v13040581

**Published:** 2021-03-30

**Authors:** Christine I. Wooddell, Adam J. Gehring, Man-Fung Yuen, Bruce D. Given

**Affiliations:** 1 Arrowhead Pharmaceuticals, 502 South Rosa Road, Madison, WI 53719, USA; bgiven@arrowheadpharma.com; 2 Toronto Centre for Liver Disease, Toronto General Hospital Research Institute, University Health Network, Toronto, ON M5G 1L7, Canada; Adam.Gehring@uhnresearch.ca; 3 Department of Medicine, The University of Hong Kong, Queen Mary Hospital, Hong Kong, China; mfyuen@hku.hk

**Keywords:** hepatitis B treatment, short interfering RNA, functional cure, HBsAg seroclearance, HBsAg reduction

## Abstract

Chronic hepatitis B infection remains a globally important cause of morbidity and mortality and has recently undergone a renaissance in therapeutic interest with increased pre-clinical and clinical testing of new drug classes. One of the first new classes in the clinic was RNA interference agents, which have the potential to impact the entire viral life cycle by reducing all virus-produced mRNA. Early clinical testing with the first of these agents in the clinic, ARC-520, demonstrated that rapid and deep reductions in viral proteins, RNA and DNA could be produced with this approach, but also the surprising insight that HBsAg production from incomplete HBV DNA integrated into the host genome appears to play a heretofore unappreciated and important role in maintaining circulating HBsAg, thought to play a fundamental role in preventing host clearance of the virus. Thus, accounting for viral DNA integration in novel HBV treatment approaches may prove to be essential to achieving successful finite therapies of this difficult to treat chronic infection.

## 1. Introduction

The hepatitis B surface antigen (HBsAg) is comprised of large, middle and small S proteins that form the envelope of the HBV virion. The three HBsAg transcripts are initially transcribed from covalently closed circular DNA (cccDNA), as are the pregenomic RNA (pgRNA) encoding core and HBV polymerase, mRNA encoding the precore protein (HBeAg) and mRNA encoding the X protein. HBsAg is also released from hepatocytes as subviral particles that do not contain viral DNA. The quantity of subviral particles produced in chronically HBV-infected individuals (CHB) can be 1000-fold greater than the number of virions. This large excess of HBsAg can also be transcribed from HBV that has integrated into the host DNA [1].

HBV integration is a by-product of the replication process and occurs when the primed DNA minus strand fails to switch strands, resulting in a double-stranded linear DNA (dslDNA) product with Direct Repeat 1 (DR1) at both ends [2]. Some nucleocapsids contain dslDNA, increasing with progression of liver disease [3]. Linear DNA is prone to integrate and tends to lose some sequences from both ends in so doing. Predecessors of the hepatitis B virus left their integrated footprints in the genomes of birds and mammals millions of years ago [4]. For a virus this evolutionarily successful, the integration of HBV is likely to be an integral player in HBV’s propagation. The dslDNA does not contain the entire viral genome and, thus, cannot replicate to produce virions, but it can produce an abundance of HBsAg.

The abundance of circulating HBsAg leads to persistent exposure to antigen, which drives exhaustion of antigen specific immunity. There is potentially no other infection where this is more relevant than chronic hepatitis B. Neonatal or early childhood infection means that the majority of CHB patients have been infected for decades, exposing HBV-specific T and B cells to high levels of both HBsAg and HBeAg. As a result, both the frequency and function of HBV-specific T cells are impaired [5]. The functional impairment is directly related to the level of circulating antigen, with HBsAg-specific T cells being more difficult to detect than HBc- or HBV polymerase (HBp)-specific T cells [6,7]. HBV-specific B cells do not suffer the same decline in frequency as T cells but the impact of antigen levels is still evident with HBs-specific B cells displaying an exhausted (PD-1+) atypical phenotype while HBcAg-specific B cells remain functional [8,9,10]. Therefore, given the direct correlations of the level of antigen on adaptive immune function, reducing peripheral exposure through therapeutics such as RNAi provides an opportunity to restore functionality.

The impact of circulating HBV antigens on innate immunity is much less clear. The data are conflicting as to whether HBV proteins directly impair innate immune cells. Many of these data are derived from in vitro systems using recombinant antigens and cells from uninfected donors. Similarly, HBV-mediated inhibition of innate signaling pathways within infected hepatocytes has been reported but remains controversial. Both topics were recently reviewed with indepth coverage of the complexity of the data [11,12]. However, observations have been made in patients demonstrating that alterations in innate immunity occur, either impacting the expression of Toll-like receptors or modulating immune cell composition in the peripheral blood [13]. Therefore, RNAi-based therapeutics present the first opportunity to definitively measure the impact of therapy-induced reductions of HBV and HBV antigens on innate immune composition and function.

If untreated, approximately 25% of individuals with CHB will die of the complications of cirrhosis and/or HCC [14,15]. Inhibition of viral replication with the highly effective nucleos(t)ide analogs (NUCs) greatly reduces these consequences of CHB but rarely leads to a “functional cure”, defined as the loss of circulating HBsAg [16,17]. Such HBsAg seroclearance occurs at a rate of 0.1–2% of CHB patients per year without treatment and in 3–11% of patients after years on NUCs [18,19]. Only a subset of patients are predicted to respond to PEGylated interferon treatments and these treatments have considerable side effects, limiting their use. Participants of the 2019 EASL-AASLD HBV Treatment Endpoint Conference agreed that the primary endpoint desired for clinical trials of new therapeutics is functional cure of CHB [20].

Arguably, the most promising approach to reduce the abundant HBsAg and other HBV proteins is RNAi, which utilizes a catalytic mechanism whereby the endogenous RNA-induced silencing complex (RISC) employs small interfering RNA (siRNA) or the synthetic equivalent to mediate cleavage of target RNA complementary to the siRNA. Hereby the target RNA and the proteins that would have been translated from it are reduced. The “siRNA” for therapeutic purposes is fully synthetic and does not contain ribonucleotides. Modifications of the ribose such as 2’ fluoro or 2’-O-methyl increase stability of the siRNA and abrogate the innate immune response that would result from use of RNA. RNAi can be utilized to reduce the pgRNA and all viral transcripts if the siRNA position is strategically selected. This includes transcripts from both the cccDNA and the integrated HBV. Thus, RNAi is a powerful approach to reduce HBV proteins and nucleic acids [21].

**Figure 1:**
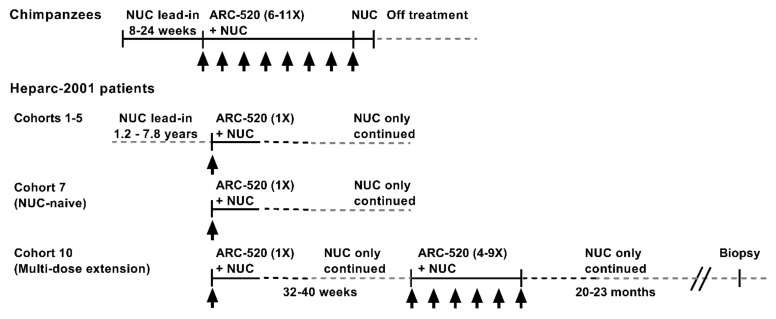
Schematic of ARC-520 study in chimpanzees and clinical trial Heparc-2001 in chronically HBV-infected patients treated with ARC-520.

The RNAi therapeutic ARC-520 was evaluated in chimpanzees chronically infected with hepatitis B and in Chinese patients with CHB that enrolled in clinical trial Heparc-2001 (NCT02065336) [1]. The treatment schematic is shown in Figure 1. Chimpanzees were treated with NUCs for a lead-in period of 8–24 weeks prior to receiving doses of ARC-520 once every 4 weeks (Q4W). Chimpanzees received 6–11 doses of ARC-520 (2–4 mg/kg) followed by 1–2 weeks of NUC alone prior to termination of all treatment.

The treatment schematics for clinical trial Heparc-2001 cohorts 1–5, 7 and 10 are shown in Figure 1. Cohorts 1–5 (*n* = 8 randomized 6:2 drug: placebo) had been on entecavir treatment for 1.2–7.8 years prior to enrolling in this study. They received a single dose of ARC-520 (1–4 mg/kg) and continued entecavir. Cohorts 1–4 were HBeAg-negative. Cohort 5 was HBeAg-positive. Cohort 7 was an open label cohort with patients that were initially NUC-naïve. Eight of the cohort 7 patients were HBeAg-negative and eight were HBeAg-positive, but one had very low HBeAg. These patients started entecavir treatment concomitant with a single dose of ARC-520 (4 mg/kg) and continued to receive entecavir for the duration of the study.

Cohort 7 patients were offered the opportunity to enroll in the multi-dose ARC-520 (4 mg/kg) extension study as cohort 10, continuing to receive entecavir. Three HBeAg-positive and five HBeAg-negative patients enrolled in the extension study reported in [22]. Thirty-two to forty weeks elapsed between the single ARC-520 dose and the multiple doses. Patients then received 4 to 9 doses of ARC-520 (Q4W). Patients continued receiving entecavir for approximately 20–23 months after the last of the multiple ARC-520 doses, at which time six of the eight patients consented to biopsy. Five of these biopsies had sufficient tissue for intrahepatic HBV DNA analysis: one from an HBeAg-positive patient and four from HBeAg-negative patients.

All cohort 10 patients in the extension study had lower HBsAg post-study than pre-study. Two of the eight patients in the extension study seroconverted for HBsAg, one initially highly viremic HBeAg-positive patient and one less viremic HBeAg-negative patient. These two patients were taken off NUCs and remained serum HBV DNA undetectable and HBsAg-negative at time of this report. Viral dynamics will be discussed.

## 2. Research Review

### 2.1. Learnings from the Development of RNAi Therapeutic ARC-520

Arrowhead Pharmaceutical’s ARC-520 was the first RNAi therapeutic in clinical trials to treat CHB. Development of ARC-520 began in 2011. Selection of the siRNA sequences and the delivery excipient to comprise an HBV RNAi therapeutic were described in [23]. HBV polymerase is an error-prone reverse transcriptase and although mutations that maintain viral fitness are constrained by the compact 3.2 kilobase (kb) HBV genome with overlapping reading frames, variations can be found at every position of the genome within the NCBI GenBank database. The first step in siRNA selection was, therefore, to identify highly conserved sequences by computational analysis. An in silico analysis was utilized to identify stretches of 17-nucleotide sequences that were at least 90% identical in each genotype. The first and last positions of a 19 base-pair siRNA are not viewed as essential for target sequence selection; thus, the sequences analyzed were antisense strand positions 2–18. Conserved sequences tended to be in regions of overlapping reading frames within the HBV genome.

Conserved siRNA sequences were synthesized with chemically modified bases to avoid triggering an off-target innate immune response and tested for activity in vitro. Of the four most efficacious siRNA sequences, two that were in the open reading frame (ORF) of the small S gene were named siHBV-75 and siHBV-76 and two that were near the 3’ terminus of the X gene ORF were named siHBV-74 and siHBV-77. These siRNAs targeted positions 380–398 (siHBV-75), 674–692 (siHBV-76), 1779–1797 (siHBV-74) and 1825–1843 (siHBV-77) of GenBank Accession #V01460.

**Figure 2:**
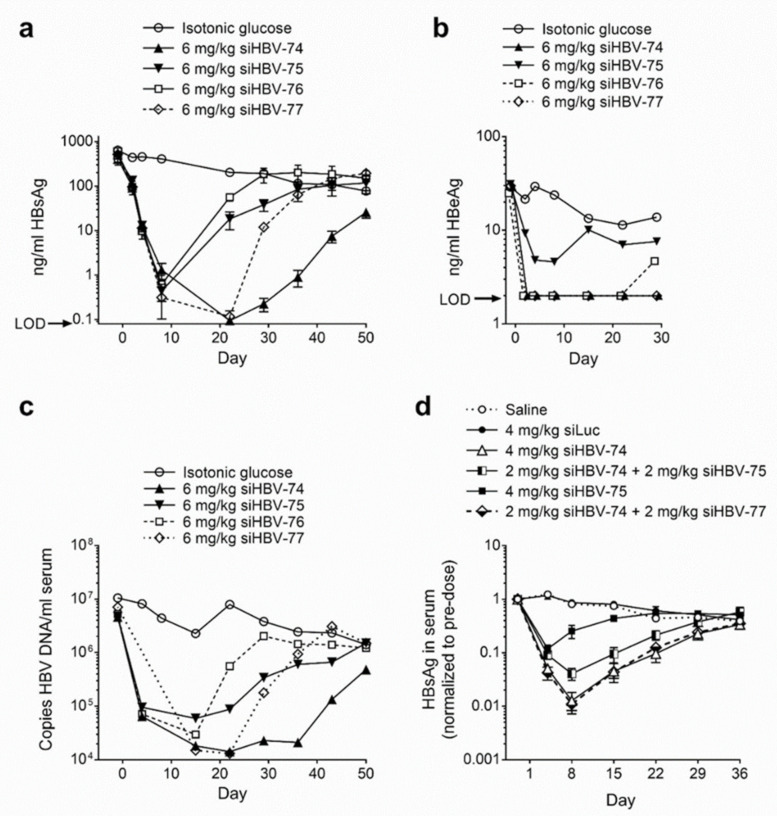
Efficacy and duration of knockdown following co-injected cholesterol-conjugated HBV siRNA and excipient NAG-MLP in mouse model of chronic HBV infection. NOD-SCID mice were given a hydrodynamic tail vein injection with (**a**–**c**) 10 μg pHBV1.3 or (**d**) 5 μg MC-HBV1.3. Three or more weeks thereafter, mice (*n* = 3–4) were given one 200 μL IV coinjection of 6 mg/kg NAG-MLP and 6 mg/kg chol-siRNA siHBV-74, -75, -76, or 77 (**a**–**c**) or 4 mg/kg NAG-MLP and 4 mg/kg chol-siRNA as indicated (**d**). (**a**,**d**) HBsAg and (**b**) HBeAg in serum were measured by enzyme linked immunosorbent assay at the indicated times relative to injection on day 1; LOD, limit of detection. (**c**) DNA was isolated from serum and the concentration of HBV genomes was quantitated by qPCR. Reproduced from [23] by permission of American Society of Gene and Cell Therapy.

The in vivo siRNA delivery approach developed by Arrowhead utilized cholesterol-conjugated synthetic siRNA (chol-siRNA) co-injected intravenously with the delivery excipient N-acetylgalactosamine-conjugated melittin-like peptide (NAG-MLP). This excipient enhanced endosomal escape of siRNA molecules that became co-localized with the MLP. Chol-siRNAs siHBV-74, siHBV-75, siHBV-76 and siHBV-77 were individually co-injected with NAG-MLP into a mouse model of chronic HBV infection to compare efficacy (Figure 2a–c). The mouse model was established by hydrodynamic injection of either a plasmid or a minicircle containing 1.3 genome lengths of HBV, referred to as the pHBV mouse model. The two most efficacious siRNAs were siHBV-74 and siHBV-77 [23].

Arrowhead opted to include two siRNAs in ARC-520 to inhibit viral escape and to provide greater genotype and genome coverage. RNAi therapeutics may be administered with NUCs that inhibit viral replication to reduce the likelihood of viral escape. Although NUCs are highly effective at reducing replication, the inhibition is not complete. Combination of siHBV-74 + siHBV-75 (1:1) and NAG-MLP was compared to that of siHBV-74 + siHBV-77 (1:1) and NAG-MLP in the pHBV mouse model (Figure 2d). The combination of siHBV-74 + siHBV-77 yielded greater HBsAg reduction than the combination of siHBV-74 + siHBV-75. In 2012, essentially all of the secreted HBsAg was thought to be produced by cccDNA, for which the pHBV mouse was a model. Therefore, siHBV-74 and siHBV-77 were selected to comprise ARC-520 along with NAG-MLP. The endosomal escape agent NAG-MLP for ARC-520 was named ARC-EX1 (EX1). The chol-siRNAs and EX1 were at a 1:1 weight:weight ratio in ARC-520.

### 2.2. Chronically HBV-Infected HBeAg-Positive and HBeAg-Negative Chimpanzees Responded Differentially to ARC-520 but Demonstrated Similar RNAi Capacity

**Figure 3:**
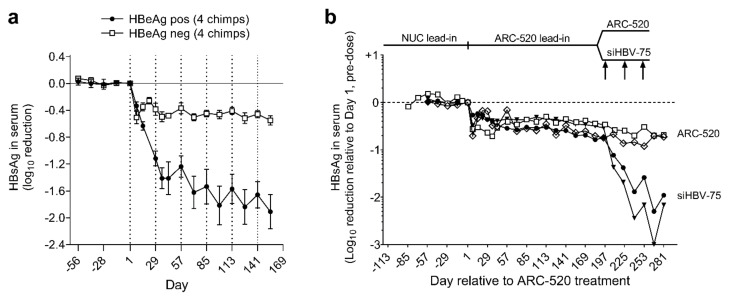
Response to repeat dosing of chimpanzees with HBV siRNAs. HBeAg-positive and HBeAg-negative chimpanzees began daily NUC treatment for a lead-in period of 8 to 24 weeks prior to commencing Q4W dosing with ARC-520, beginning on Day 1. HBsAg reduction is shown relative to Day 1 of ARC-520 treatment. (**a**) HBsAg reduction following 6 doses of ARC-520 in HBeAg-positive and HBeAg-negative chimpanzees. (**b**) Four HBeAg-negative chimpanzees received 7 doses of ARC-520. Then, two chimpanzees received three additional doses of ARC-520 while two animals received three doses of siHBV-75 + EX1. Reproduced from [1]. Reprinted with permission from AAAS.

Simultaneously with a clinical study discussed below, the efficacy of ARC-520 was evaluated in nine chimpanzees, five males and four females as described in [1]. Four chimpanzees were HBeAg-negative and five chimpanzees were initially HBeAg-positive, but one had a very low amount of HBeAg and was transitioning to become HBeAg-negative. After a lead-in period of daily oral NUCs, HBeAg-positive and HBeAg-negative chimpanzees received six or more intravenous injections of ARC-520, once every four weeks (Q4W) and continued NUCs. After six injections, HBsAg was reduced by a mean −1.91 log_10_ in four HBeAg-positive chimpanzees but only by −0.55 log_10_ in four HBeAg-negative chimpanzees (Figure 3a).

The differential response in HBeAg-positive compared to HBeAg-negative chimpanzees was unexpected and was examined by analysis of their HBV transcripts, isolated from periodic liver biopsies, by three different approaches: reverse transcription Real-Time qPCR (RT-qPCR), paired-end next-generation mRNA sequencing (mRNA-seq), and single-molecule real-time (SMRT) sequencing. RT-qPCR revealed that the HBeAg-positive chimpanzees had a mean 18-fold more total HBV transcripts than the HBeAg-negative chimpanzees, consistent with the higher amounts of HBsAg, viral DNA and HBeAg produced in the HBeAg-positive chimpanzees. In the HBeAg-negative chimpanzees only 3.5 ± 0.5% of the total HBV transcripts (measured with a probe in the X gene) were pgRNA + pre-core (measured with a probe in the core region), whereas 52.6 ± 0.5% of the total transcripts were pgRNA + pre-core in the HBeAg-positive chimpanzees. While interesting, this finding was not surprising because these HBeAg-negative chimpanzees maintained serum HBV DNA levels near the lower level of quantitation (LLOQ) prior to any treatment. 

**Figure 4:**
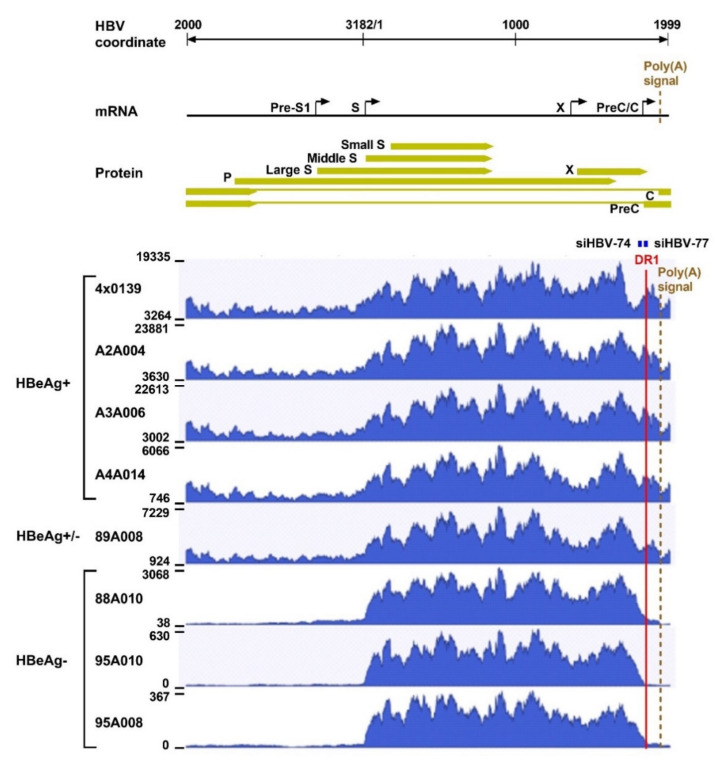
Liver HBV mRNA paired-end sequencing reads in HBeAg-positive and HBeAg-negative chimpanzees treated with ARC-520. The HBV mRNA and HBV protein open reading frames are positioned relative to the coordinates of the HBV genome. The mRNA-seq read histograms are shown for HBeAg-positive chimpanzees 4 × 0139, A2A004, A3A006, and A4A014; for HBeAg transitional chimpanzee 89A008; and for HBeAg-negative chimpanzees 88A010, 95A010, and 95A008. Locations of the DR1 sequence (red line), HBV PAS (brown dashed line), and binding sites for the siRNAs in ARC-520 (siHBV-74 and siHBV-77) are indicated. From [1]. Reprinted with permission from AAAS.

**Figure 5:**
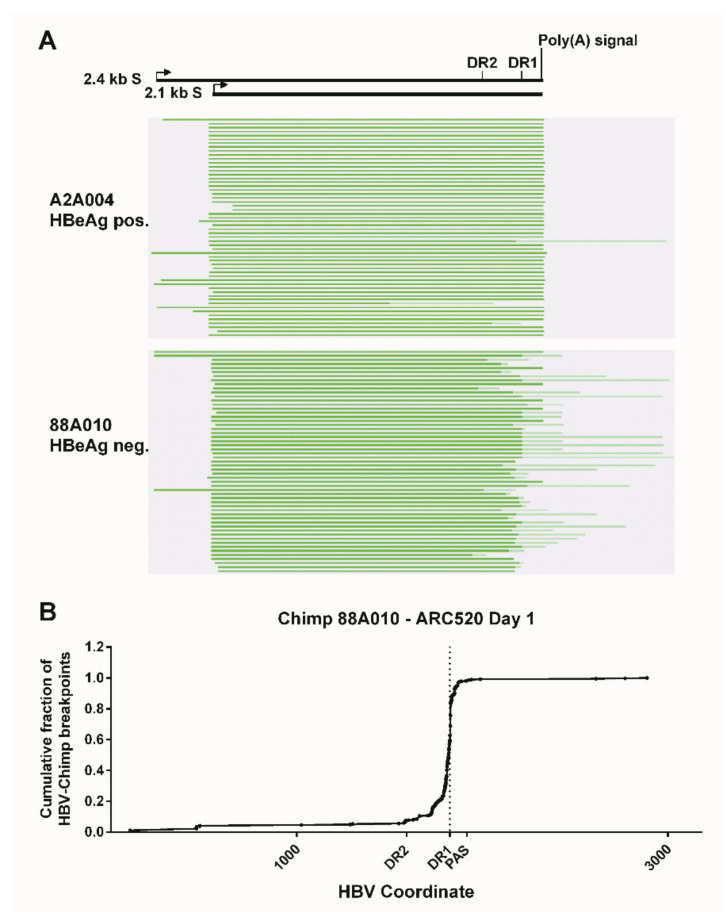
Mapping of HBV S transcripts from HBeAg-positive and HBeAg-negative chimpanzees. Total RNA isolated from the liver biopsies after the NUC lead-in and before ARC-520 dosing on day 1 from HBeAg-positive chimpanzee A2A004 and HBeAg-negative chimpanzee 88A010 was reverse-transcribed, size-selected, and sequenced with SMRT sequencing. (**A**) Full-length nonconcatemer reads were aligned to each chimpanzee’s consensus HBV DNA sequence. Green lines represent HBV containing transcripts. Dark green represents sequences aligning to HBV, and light green represents those not aligning to HBV. The HBV coordinates are shown with elements DR2, DR1, and the HBV polyadenylation signal. (**B**) Cumulative fraction of HBV-chimpanzee breakpoints was plotted against the HBV coordinate. From [1]. Reprinted with permission from AAAS.

What was surprising when first observed was that most HBV transcripts in the HBeAg-negative chimpanzees did not extend to the HBV polyadenylation signal, whereas those in HBeAg-positive chimpanzees did (Figure 4). Far fewer HBV transcripts in HBeAg-negative chimpanzees, relative to the HBeAg-positive chimpanzees, appeared to include the location of the binding sites for siHBV-74 and siHBV-77. This observation was the first hint that HBsAg was being produced from integrated HBV. The promoters for the S genes and their entire ORFs are well within the dslDNA that has DR1 at either end. The key feature that could be lacking in S transcripts produced from integrated HBV is the polyadenylation signal to terminate the mRNA, although an alternate polyadenylation signal upstream of DR1 had been reported [24]. Definitive proof that S transcripts originated within the integrated HBV was obtained by SMRT sequencing of full-length transcripts from two HBeAg-positive and two HBeAg-negative chimpanzees. Aligned sequences from one HBeAg-positive and one HBeAg-negative chimpanzee are shown in Figure 5. HBV S transcripts of HBeAg-positive chimpanzees terminated primarily at the regular HBV polyadenylation signal, whereas most S transcripts of HBeAg-negative chimpanzees included chimpanzee genomic sequence at the 3’ ends and terminated at a cryptic polyadenylation signal within the chimpanzee sequence. A smaller fraction of the S transcripts terminated at the alternate polyadenylation signal, which did not allow determination of their origin, whether from cccDNA or integrated HBV.

The chol-siRNA siHBV-75 with its target site in the S ORF was employed to test the hypothesis that HBsAg was being produced from integrated HBV in HBeAg-negative chimpanzees. Four HBeAg-negative chimpanzees were each given seven Q4W injections of ARC-520 (2–4 mg/kg). Then, two of these chimpanzees were given an additional three injections of ARC-520 (4 mg/kg) while the other two were given three Q4W injections of chol-siHBV-75 (4 mg/kg) co-injected with EX1 (4 mg/kg) as shown in Figure 3b. Following the final injection, HBsAg had decreased by -0.7 log_10_ in the two chimpanzees that received ARC-520 but was reduced by −1.4 and −1.7 log_10_, −1.9 and −2.4 log_10_, and finally −2.3 and −3.0 log_10_ relative to day 1 after each of the three successive siHBV-75 doses in the other two chimpanzees. These results demonstrated that RNAi was just as effective in HBeAg-negative chimpanzees as it was in HBeAg-positive chimpanzees when the siRNA target sequence was in the RNA.

### 2.3. HBeAg-Positive Chimpanzee with Reduced Off-Treatment HBV Parameters

**Figure 6:**
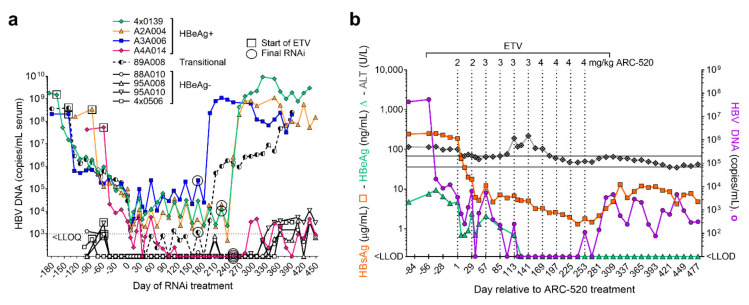
Chimpanzee response to NUC plus ARC-520. (**a**) HBeAg-positive, HBeAg-transitional and HBeAg-negative chimpanzees began daily NUC treatment (box) for a lead-in period of 8 to 24 weeks prior to commencing Q4W dosing with ARC-520, beginning on Day 1. Timing of the final siRNA injection, either ARC-520 or siHBV-75 + EX1 is indicated by a circle. NUC treatment ended approximately one week after the final siRNA injection. Serum HBV DNA levels are shown relative to Day 1 of ARC-520 treatment. (**b**) HBsAg, HBeAg and ALT levels (left *y*-axis) and serum HBV DNA (right *y*-axis) are shown for chimpanzee A4A014 prior to treatment, during entecavir lead-in, during entecavir + ARC-520 treatment, and then off all treatment. The normal ALT range for chimpanzees is shown by grey horizontal lines.

NUC treatment of the chimpanzees ended approximately one week after the last RNAi injection. Biweekly serum HBV DNA measurements revealed that replication resumed in all chimpanzees after RNAi suppression abated and NUC treatment stopped (Figure 6a). Serum HBV DNA returned to the pre-study levels in all animals except chimpanzee A4A014. 

Prior to any treatment, chimpanzee A4A014 was HBeAg-positive with 7.74 log_10_ copies/mL serum HBV DNA and 2.4 log_10_ µg/mL HBsAg. ALT levels were elevated (98–117 U/L) pre-study and during the 8-week NUC lead-in but decreased during the initial ARC-520 treatment as HBsAg decreased (Figure 6b). ALT then increased to 218 U/L following the fifth dose of ARC-520, after which this chimpanzee seroconverted for HBeAg. The nadir of HBsAg reduction was −2.3 log_10_ µg/mL. Replication was undetectable for 17 weeks while this chimpanzee was being treated with ARC-520 and NUCs. After termination of ARC-520 and NUCs, serum HBV DNA increased to 3.66 log_10_ copies/mL and then began to decrease in the absence of any treatment. Similarly, HBsAg was reduced to 0.12 log_10_ µg/mL on treatment, increased to 1.11 log_10_ µg/mL after termination of all treatment, and then decreased off treatment. Seven months after the end of all treatment, this chimpanzee was HBeAg-negative, serum HBV DNA was <LLOQ, HBsAg was 0.68 log_10_ µg/mL, liver HBV RNA was 99% lower than pre-treatment and ALT was 45 U/L (in the normal range for uninfected chimpanzees). This chimpanzee appears to have transitioned to the immune control or low replicative phase of chronic infection, a step in the direction toward control of the virus although short of HBsAg seroclearance.

The levels of serum HBV DNA continued to decline over the treatment period in most of the HBeAg-positive chimpanzees but had not become undetectable in any but A4A014 (Figure 6a). The transitional chimpanzee (ID 89A008) became HBeAg-negative but did not maintain reduced amounts of serum HBV DNA nor HBsAg off treatment, despite a delayed return to baseline for these two parameters. HBV DNA in this chimpanzee was undetectable at only a few time points during treatment. The chimpanzees with the highest amounts of replication received a shorter period of ARC-520 treatment because they were pre-treated with NUC alone for a longer period of time, relative to the other chimpanzees, and the study period ended at roughly the same time in all chimpanzees. In summary, only one out of five chimpanzees that were initially HBeAg-positive demonstrated alterations in all HBV parameters off treatment and this was the animal in which serum HBV DNA was undetectable for multiple months while on treatment. 

The four chimpanzees that were HBeAg-negative pre-study had serum HBV DNA that fluctuated near the LLOQ, prior to and after treatment. During the 19-month study, ALT was in the normal range for two of these chimpanzees and fluctuated between the normal range and up to 1.5× upper limit of normal in the other two chimpanzees. The normal ALT range for chimpanzees without HBV infection where most were housed was 30–95 U/L, possibly due to some being obese, and the range was not adjusted for sex. Although these HBeAg-negative chimpanzees had integrated HBV producing the majority of the HBsAg, the active albeit low level of replication indicates they must also have had cccDNA. The two chimpanzees treated with siRNA able to target S mRNA produced from integrated HBV received only a few doses, limiting our understanding of host response to long-term RNAi suppression of HBsAg in HBeAg-negative hosts.

### 2.4. Chronically HBV-Infected Patients Treated with ARC-520

**Figure 7:**
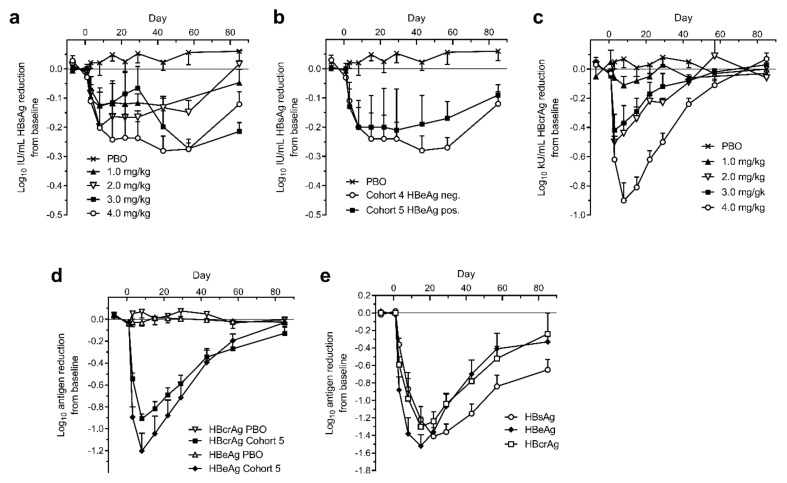
Serum HBsAg, HBcrAg and HBeAg in human patients treated with a single dose of ARC-520. CHB patients were given a single intravenous dose of ARC-520 (1 to 4 mg/kg) on a background of daily oral NUCs. HBsAg (**a**) or HBcrAg (**c**) reduction in CHB patients who were NUC-experienced HBeAg-negative and received single doses (1 to 4 mg/kg) (cohorts 1 to 4, *n* = 6). (**b**) HBsAg reduction in CHB patients who were NUC-experienced HBeAg-negative (cohort 4, *n* = 6) or NUC-experienced HBeAg-positive (cohort 5, *n* = 6) who received a single dose (4 mg/kg). (**d**) HBcrAg and HBeAg reduction in CHB patients who were NUC-experienced HBeAg-positive and received a single dose (4 mg/kg) (cohort 5, *n* = 6). (**e**) HBsAg, HBeAg, and HBcrAg reductions in CHB patients who were NUC-naïve HBeAg-positive and received a single dose (4 mg/kg) (cohort 7, *n* = 5). Reproduced from [1]. Reprinted with permission from AAAS.

A single intravenous injection of ARC-520 was administered to chronically HBV infected patients in the phase 2 clinical trial Heparc-2001 [1]. Patients in Cohorts 1 to 5 had been on NUC therapy for 1.2 to 7.8 years prior to this study and continued daily NUC treatment during the study. Cohorts 1 to 4 enrolled HBeAg-negative patients with serum HBV DNA <LLOQ and these patients received 1, 2, 3 or 4 mg/kg ARC-520, respectively, and continued NUCs. Cohort 5 patients were also NUC experienced and received 4 mg/kg ARC-520 along with continued NUCs, but they were HBeAg-positive. HBsAg was only modestly reduced in the cohort 1 to 5 patients that were NUC-experienced, whether HBeAg-negative or HBeAg-positive (Figure 7a,b). HBsAg was reduced by -0.3 log_10_ IU/mL in patients that received 4 mg/kg ARC-520. This reduction was substantially less than in the mouse model.

The reduction of HBV core-related antigen (HBcrAg) in the HBeAg-negative patients of cohorts 1 to 4, however, was substantially greater than the HBsAg reduction and consistent with the administered dose of ARC-520 (Figure 7c). Cohort 4 patients reached −0.9 log_10_ kU/mL mean HBcrAg reduction. Similar reductions of −0.9 log_10_ kU/mL HBcrAg and −1.2 log_10_ PEI U/mL HBeAg were observed in HBeAg-positive cohort 5 (Figure 7d). These results demonstrated that ARC-520 had similar efficacy in HBeAg-negative and HBeAg-positive patients when targeting cccDNA-derived pgRNA and when targeting pre-core transcripts in the NUC-experienced HBeAg-positive patients.

HBeAg-positive and HBeAg-negative CHB patients that were NUC-naïve at start of study were enrolled in cohort 7 of Heparc-2001. They received a single 4 mg/kg dose of ARC-520 and began receiving daily NUCs. Excluding one patient who had low HBeAg, the previously NUC-naïve HBeAg-positive patients in this cohort responded with deep reductions of HBsAg: a mean of −1.4 ± 0.1 log_10_ IU/mL (minimum of −1.3 log_10_ and a maximum of −1.8 log_10_ reduction), along with mean −1.5 ± 0.1 log_10_ PEI U/mL reduction in HBeAg and −1.3 ± 0.1 log_10_ kU/mL reduction in HBcrAg (Figure 7e). Maximal HBsAg reduction in the HBeAg-negative patients of cohort 7 was the same as in the NUC-experienced patients of cohorts 4 and 5 (data not shown). Thus, ARC-520 mediated deep reduction of HBsAg, HBeAg and HBcrAg in the subset of CHB patients that were HBeAg-positive and NUC-naïve. 

### 2.5. Accelerated Reduction of HBV Parameters in Patients Treated with Multiple Doses ARC-520 Plus NUC

**Figure 8:**
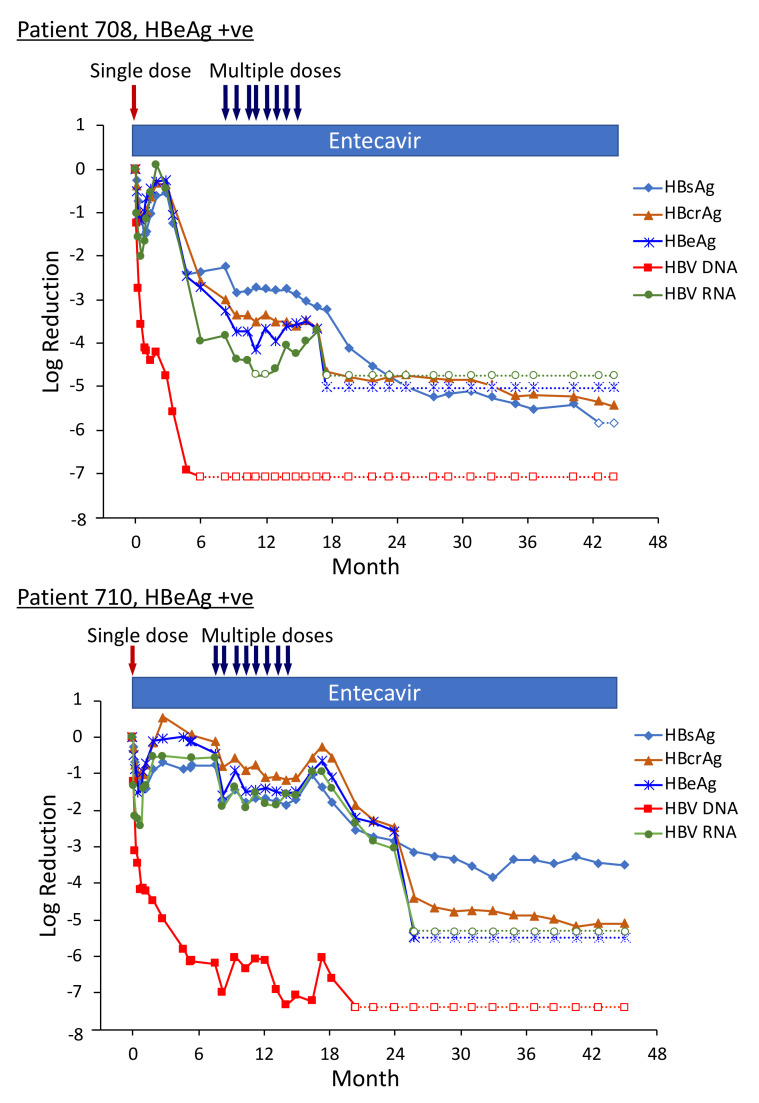

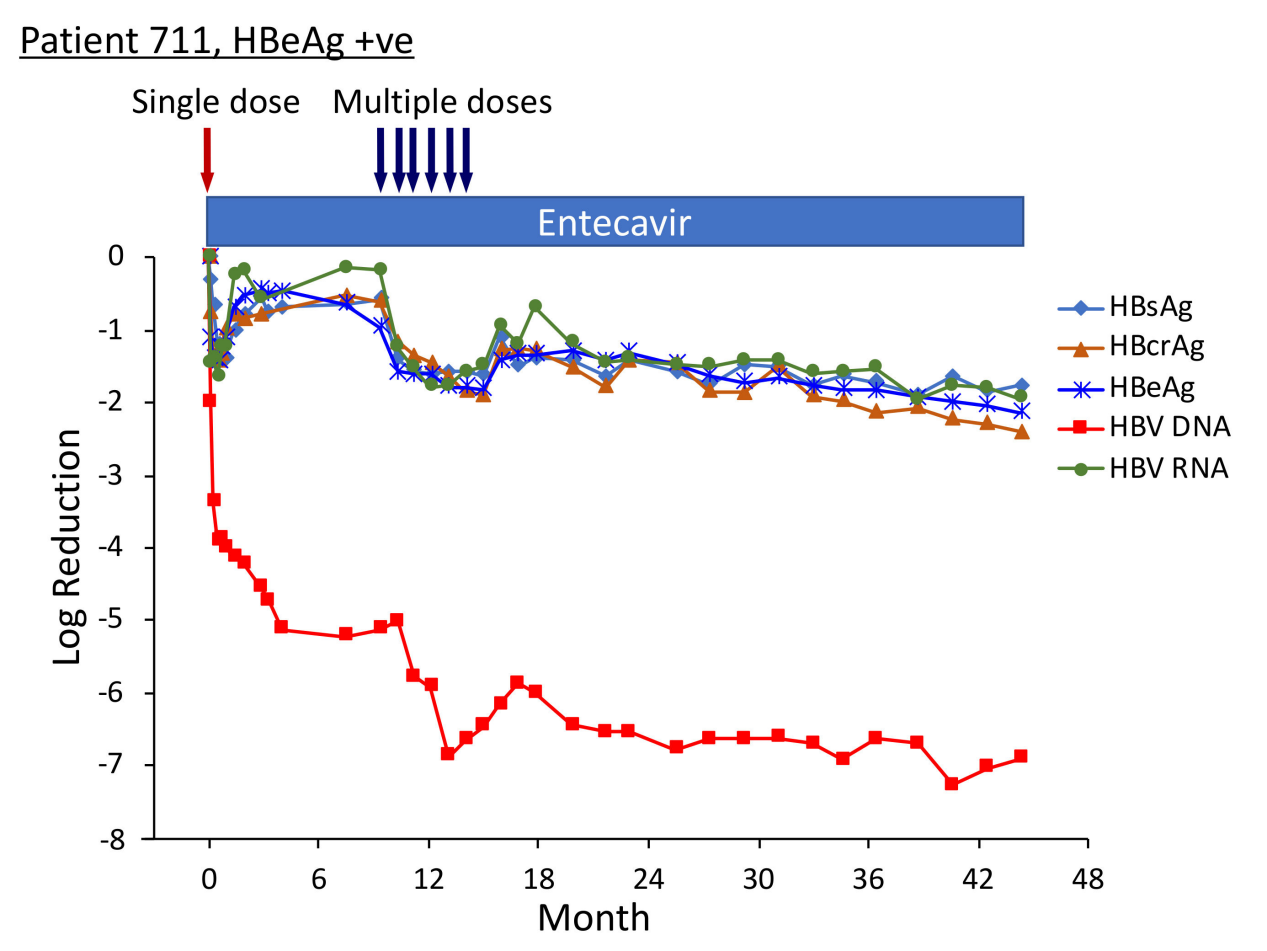
HBV parameters in HBeAg-positive patients treated with multiple doses of ARC-520. Previously NUC-naïve patients 708, 710 and 711 received daily NUC beginning on study Day 1, a single dose of ARC-520 on Day 1, and then multiple doses of ARC-520 (indicated with arrows) beginning approximately 7 months after the single dose of ARC-520. Log_10_ reductions in HBsAg, HBcrAg, HBeAg, serum HBV DNA and serum HBV RNA are shown. Empty symbols indicate the analyte was not detected. Reproduced from [22] with permission from BMJ Publishing Group Ltd. of the British Medical Association, London, UK.

Three of the HBeAg-positive patients from Heparc-2001 cohort 7 were enrolled as cohort 10 into an extension study to receive multiple doses of ARC-520 and continued NUC [22]. Thirty-two to 40 weeks elapsed between administration of the single ARC-520 dose to the HBeAg-positive patients in cohort 7 prior to their receiving multiple doses of ARC-520 as cohort 10. Fluctuations in the HBsAg levels during this period were of particular interest (Figure 8). The nadir of HBsAg reduction following the first dose of ARC-520 while these patients were in cohort 7 was on weeks 3–4. After nadir, the HBsAg suppression gradually diminished until week 12. In each of these patients, the HBsAg was at this time −0.5 log_10_ IU/mL to −0.7 log_10_ IU/mL lower than pre-study and the amounts did not increase further in the additional 20–28 weeks prior to the multi-dose phase of ARC-520 treatment. Rather than returning toward baseline, HBsAg in patient 708 began once more to decline while she continued NUC treatment but not ARC-520. Her HBsAg was −2.25 log_10_ IU/mL lower prior to receiving multi-dose ARC-520 than the pre-study value. HBsAg levels in patients 710 and 711 were −0.77 log_10_ IU/mL and -0.56 log_10_ IU/mL lower than the pre-study value prior to receiving multi-dose ARC-520.

Pre-study, HBsAg was 4.54 log_10_ IU/mL in patient 708; 4.91 log_10_ IU/mL in patient 710; and 4.81 log_10_ IU/mL in patient 711. At the final follow-up three years after the start of study Heparc-2001, HBsAg had not returned to baseline. HBsAg was <LLOQ in patient 708 and she was anti-HBs positive; HBsAg was 1.4 log_10_ IU/mL in patient 710 and 3.0 log_10_ IU/mL in patient 711 [22].

Serum HBV DNA levels in the three cohort 10 HBeAg-positive patients 708, 710 and 711 were 8.54 log_10_, 8.85 log_10_ and 8.69 log_10_ IU/mL prior to their enrollment in Heparc-2001. Serum HBV DNA became <LLOQ prior to NUC + ARC-520 multi-dose treatment in patient 708; but in patient 710 remained detectable during multi-dose treatment and only became <LLOQ ten months after the last of the multi-dose ARC-520 treatments; and in patient 711 serum HBV DNA did not reach <LLOQ during the three year Heparc-2001 study (including the extension) (Figure 8). There appeared to be an association between the effectiveness of serum HBV DNA decrease in response to NUC plus ARC-520 and the three-year HBsAg reductions.

**Figure 9:**
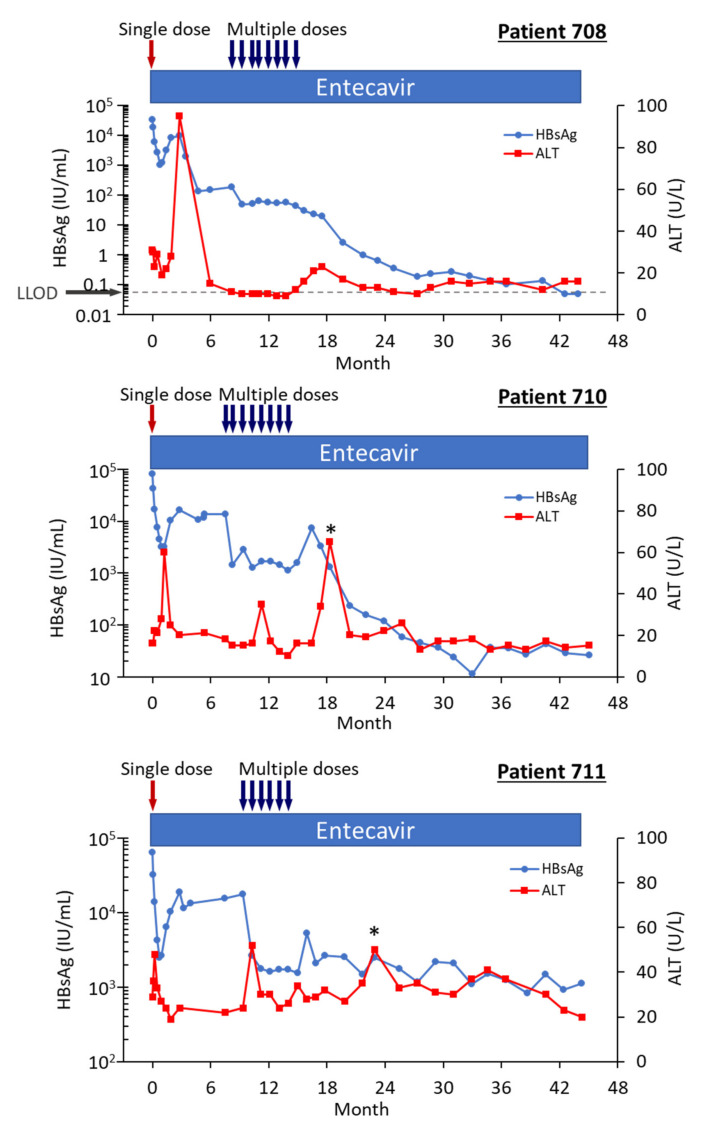
HBsAg and ALT in HBeAg-positive patients treated with multiple doses of ARC-520 in combination with NUCs. Patient 708, 710 and 711 received daily NUC beginning on study Day 1, a single dose of ARC-520 on Day 1, and then multiple doses of ARC-520 (indicated with arrows) beginning approximately 7 months after the single dose of ARC-520. Shown are the measured values for HBsAg and ALT for each timepoint. Reproduced from [22] with permission from BMJ Publishing Group Ltd., London, UK.

Highly viremic patient 708 was, thus, an interesting case study that demonstrated the potential of RNAi therapeutics [22]. Pre-study this patient had 8.54 log_10_ IU/mL serum HBV DNA, 4.54 log_10_ IU/mL HBsAg, 3.02 PEIU/mL HBeAg, 5.58 kU/mL HBcrAg and 6.39 log_10_ copies/mL HBV RNA in serum (Figure 8). Following the first dose of ARC-520, HBsAg was reduced by −1.58 log_10_ IU/mL, HBeAg was reduced by −1.2 log_10_ PEI U/mL, HBcrAg was reduced by −1.11 log_10_ kU/mL and serum HBV RNA was reduced by −2.16 log_10_ U/mL. After HBsAg began to increase following the nadir at week 3, HBsAg reached its highest post-dose value at week 12, at which point in time ALT increased to 95 U/L (Figure 9). By the next measurement approximately 3 weeks later, HBsAg, HBeAg, HBcrAg and HBV serum RNA had all begun to precipitously decrease without additional siRNA exposure and ALT had normalized. HBsAg decreased 50.6-fold between week 12 and week 35 when this patient received her first of the multiple ARC-520 doses as cohort 10. ALT remained close to 10 U/L prior to each of the 8 doses that were approximately monthly and then rose just to 23 U/L twelve weeks after the final dose of ARC-520. Following this very modest ALT elevation in the normal range for healthy women, HBsAg continued to decline until it became undetectable and the patient became anti-HBs positive. She was subsequently taken off NUC without reappearance of serum HBV DNA and HBsAg at the time of writing, i.e., 8 weeks after NUC termination. These results strongly suggest that the host was responding to increases in viral antigens (the patient was still on NUCs) and further suggests that host control of the virus does not require large-scale killing of hepatocytes, as the ALT increases were modest.

Patient 708 did not have a biopsy at the end of the study, but patient 710 did [22]. Patient 710’s HBsAg was 80,918 IU/mL pre-study and 11.5 IU/mL at the final follow up more than two years after the last ARC-520 dose. Serum HBV RNA was 9.96 log_10_ pre-study and <LLOQ at the final follow up. Upon histological assessment of patient 710’s liver, less than 5% of the hepatocytes were positive for HBsAg. Interestingly, this patient had 3.4 copies cccDNA/cell in the liver, but only 0.09 copies/cell of pgRNA. These data suggest transcription silencing of cccDNA by the end of the study.

Five HBeAg-negative patients were also enrolled in the Heparc-2001 extension study. HBsAg reduction was more modest in the HBeAg-negative patients treated with multiple doses of ARC-520 than in the HBeAg-positive patients. Even so, HBsAg decreased at nadir by approximately −0.5 log_10_ in two patients, −1 log_10_ in two patients, and by greater than 2 log_10_ in the fifth patient that achieved HBsAg seroclearance [22]. All these patients had lower HBsAg at the end of the study than pre-study four years earlier.

As in HBeAg-positive patient 708, HBsAg in HBeAg-negative patient 709 began to return toward baseline after the first dose of ARC-520, but then declined again prior to multi-dosing (data not shown). Following the nadir after five ARC-520 doses in the multi-dosing period, the HBsAg began to increase and then in the absence of ARC-520 began once more to decline until HBsAg was undetectable. Patient 709 seroconverted for HBsAg twenty-six months after start of study when the first ARC-520 dose was received and ceased entecavir treatment one year after seroconversion. Pre-study, patient 709’s serum HBV DNA had been 3.76 log_10_ IU/mL and serum HBV RNA had been 2.05 log_10_ U/mL. At the last follow-up, serum HBV RNA and serum HBV DNA were not detected. None of the hepatocytes in the liver biopsy were HBsAg-positive. This patient had 0.263 copies cccDNA/cell and 0.096 copies pgRNA/cell. At start of study patient 709 was 45 years old and had normal ALT (25 U/L).

### 2.6. Consideration of Treatment Age and CHB Phase

The AASLD 2018 guidelines recommend treating patients with NUCs when ALT >2 X ULN, and HBV DNA is >20,000 IU/mL for HBeAg-positive or >2000 IU/mL for HBeAg-negative when there is significant fibrosis, family history of HCC or patients are more than 40 years old.

Chimpanzees are not patients but studying HBV infection in these animals has provided critical insights [25]. Setting aside the last criteria regarding fibrosis (none of the chimpanzees given biopsies had fibrosis) and the unknown family history of HCC, no chimpanzees were older than 40 human years. They ranged in age from 9–34 years old at the start of study. The equivalent age in human years is unknown, but chimpanzee A4A014 who transitioned to become an inactive carrier following NUC plus ARC-520 treatment was nine years old.

The HBeAg-positive, NUC-naïve patients that were enrolled in the Heparc-2001 study would formerly be classified as “immune tolerant” and would not meet the AASLD guidelines to be treated with the currently available therapeutics: NUCs and PEGylated interferons. The purpose of clinical trial Heparc-2001 was to assess both safety and anti-viral activity of ARC-520 in patients; therefore, the three HBeAg-positive patients in the extension study all had nearly normal ALT levels (31, 16, and 29 U/L, respectively, for patients 708, 710 and 711). All three were highly viremic and less than 40 years old. Female patient 708, male patient 710 and female patient 711 were 31, 23 and 36 years old, respectively. Treatment is not currently recommended for this group primarily because no available treatments have been proven effective. However, these patients responded to ARC-520 plus NUC treatment with significant durable reductions of HBsAg, HBeAg, HBcrAg and HBV RNA following ARC-520 treatment while still on NUCs.

The initially high viremia and greater than one log decline in HBsAg following the first dose of ARC-520 in human patients 708, 710 and 711 suggest a substantial amount of their HBsAg was derived from cccDNA and not integrated HBV. These patients less than 40 years old could benefit greatly if treatment were available to prevent the ongoing integration of HBV that increases their long-term HCC risk [26,27]. Svicher et al. recently demonstrated that highly viremic CHB patients not only have many HBV integration events, but these events also disrupt normal gene expression and promote carcinogenesis [27]. Such patients have not historically responded to treatment with NUC alone or with interferon therapies; thus the exclusion in guidelines. These patients did, however, respond to the RNAi therapeutic ARC-520 plus NUC to result in lowered HBsAg by orders of magnitude after the RNAi treatment ended. There were only three initially NUC-naïve and HBeAg-positive patients that participated in the extension study, but each of them had lower HBsAg after treatment. Forty-four months after the start of the initial Heparc-2001 study, the HBsAg levels of patients 708, 710 and 711 were reduced more than −5.8 log_10_ IU/mL (HBsAg seroconversion), −3.5 log_10_ IU/mL and −1.76 log_10_ IU/mL, respectively. HBeAg, HBcrAg and serum HBV RNA, all of which are products of the cccDNA, were also reduced orders of magnitude by this time point. The final ARC-520 dose was 30 months prior to this. No therapeutics have reported reduction in HBsAg, HBeAg nor HBcrAg to this degree. Patient 708 who seroconverted for HBsAg has remained HBsAg and serum HBV DNA negative off all treatment. The other two patients have continued NUC treatment.

Clinical development of ARC-520 was discontinued following a finding due to the EX1 excipient in an animal toxicology study; however, RNAi therapeutics without an endosomal escape agent currently in clinical testing include ARO-HBV/JNJ-3989, VIR-2218, ARB-729 and RG6346.

### 2.7. Durable HBV Parameter Reductions from RNAi Therapeutic in Development

ARO-HBV/JNJ-3989 is a next generation RNAi therapeutic for CHB that is highly efficacious for HBeAg-positive patients and HBeAg-negative patients [28]. ARO-HBV/JNJ-3989 was designed to target HBsAg produced from both cccDNA and integrated HBV. Following three monthly doses of ARO-HBV/JNJ-3989 in conjunction with daily NUC treatment, 38% (*n* = 15) of the patients responded by maintained at least one log reduction of HBsAg for approximately one year (to day 392), a mean of approximately −2 log_10_ IU/mL HBsAg reduction. Some of these patients maintained a reduced level or continued to decrease their levels of serum HBV RNA, HBeAg and HBcrAg as well, all three of these being markers of cccDNA expression. These results with ARO-HBV/JNJ-3989, thus, resemble the continuing decline of HBV parameters off RNAi treatment following dosing of ARC-520 in the Heparc-2001 study. Patients continued NUC treatment in both studies, but the continued decline in viral parameters is not a typical feature of treatment with NUC alone. Understanding the mechanism of this response will require further study.

### 2.8. Efforts to Accelerate Functional Cure

In recent years multiple studies have evaluated the termination of NUC treatment as a means to increase functional cure. After stopping NUC treatment, 10–30% of patients became inactive carriers but the majority of these still had low-grade viremia [29]. CHB patients with low HBV DNA, low HBsAg and low cccDNA are currently the candidates considered to have the best prospects for functional cure upon termination of NUC treatment. These patients tend to be older. However, hepatocellular carcinoma risk may not decrease in patients who seroconvert for HBsAg after age 50, particularly if they already have advanced liver disease [30].

Relapse was observed to be lower in patients who had consolidation therapy for at least 11 or 15 months after HBeAg seroconversion [31,32]. Lower levels of HBV DNA during treatment correlated with a lower rate of relapse [33]. These studies are consistent with our observations of a more effective response in the Heparc-2001 patients who had a longer period of well-suppressed HBV DNA on NUC treatment.

The relapse rate in HBeAg-positive CHB patients that had become HBeAg-negative was observed to be lower in patients who were less than 37 or 40 years old when they seroconverted for HBeAg [31,32]. It remains to be determined if the sustained response post-RNAi treatment will vary by age. Overall, T cell responses do not appear different across the different stages of chronic HBV infection [34]. However, specific analysis of younger patients has demonstrated potentially broader T cell function compared to older patients [35], providing a testable hypothesis for future clinical studies. In any case, the ability of RNAi to produce deep reductions in viral antigens, even HBsAg from integrated DNA, and possibly added benefits from combination with other new therapeutic classes should allow testing in patients across the broad biological spectrum of chronic HBV infection and employing novel stopping or treatment consolidation strategies in the continued search for higher rates of functional cure.

## 3. Conclusions

Functional cure by definition is loss of HBsAg expression. Due to the majority of HBsAg being expressed from integrated HBV in HBeAg-negative CHB, functional cure almost certainly means loss of hepatocytes with integrated HBV concomitant with prevention of new infection. The mechanism of continued HBsAg decline post-RNAi treatment is of great interest and creates the opportunity to address critical questions related to the objective of HBV cure. The ability to reduce viral antigens in vivo will resolve the conflicting data regarding the impact of HBsAg on innate immunity. Likely more important is addressing the impact of HBsAg reduction on HBV-specific T cell functional restoration. Recent evidence from a cross-sectional study suggests that T cell recovery is limited in the absence of antigen, even in patients that experience functional cure [36]. However, it is difficult to envision other mechanisms capable of the prolonged, post-treatment responses observed in the Heparc-2001 study [22]. Therefore, it is important that future RNAi clinical trials incorporate an immunological component capable of characterizing the HBV-specific T cell response.

While T cell restoration provides an attractive hypothesis for the maintenance of HBsAg reduction after RNAi therapy, the antiviral mechanism of CD8 T cells is counterintuitive to the mechanism of action for RNAi. RNAi shuts down HBsAg production at the source, limiting hepatocyte antigen presentation that exhausts antigen specific T cells [37,38,39,40]. However, hepatocytes are poor antigen presenting cells and reducing antigen production in hepatocyes will also reduce their visibility to CD8 T cells [41]. It is possible that the gradual rebound in HBsAg allows for a prolonged period of recognition and clearance by recovered CD8 T cells. This can be tested and it may turn out that elimination of hepatocytes with HBsAg integration is not due to conventional CD8 T cell recognition, particularly because it was not associated with significant ALT elevation. Alternatively, reduction in hepatocyte presentation using RNAi has already shown benefits in combination therapy in preclinical mouse models, boosting T cell immunity in RNAi treated animals with therapeutic vaccines [42]. Therefore, in addition to their potent direct acting antiviral mechanism of action, RNAi therapeutics show great promise in combination with immunotherapies in eliminating both infected and integrated hepatocytes to increase rates of functional cure.

The post-RNAi treatment reduction of HBeAg, HBcrAg and HBV RNA in initially HBeAg-positive patients with high viremia indicates expression from cccDNA was reduced. This reduction could be due to cccDNA loss, to silencing of expression or both [43]. The episomal HBV cccDNA is much like plasmid DNA. Nucleosomes form on episomal DNA and can result in chromatinization and silencing [44]. Chromatinization of HBV cccDNA being expressed differs from that of chromosomal DNA or plasmid DNA in that core protein forms part of the nucleosomes in HBV cccDNA [45]. The decay of core proteins on the cccDNA could be part of the mechanism for cccDNA silencing following extended RNAi treatment. Interestingly, Cheng et al. recently demonstrated that reduced HBx protein expression results in the establishment of a repressed chromatin state [46]. ARC-520 is expected to cleave both X mRNA and core-encoding RNA (pgRNA). The mechanism whereby replication returns to baseline levels following the end of a treatment has been called “replication memory.” Further study is required to understand if this replication memory is the result of reactivated cccDNA following alleviation of RNAi-suppressed core and X protein expression, for example. A preferable result of treatment would be loss of cccDNA, but this step is slow [43].

Cell division can result in cccDNA loss. Large-scale cell killing in acute HBV infection results in cell division of a large percentage of the hepatocytes and plays a role in loss of cccDNA during resolution of acute infection. In large-scale hepatocyte loss such as from partial hepatectomy of mice or resection of liver in human patients, hepatocytes of the parenchyma divide to replenish the liver. In diseases with chronic low-level hepatocytes loss, hepatocytes are replenished from the oval cells [47,48,49,50]. The low ALT in the HBeAg-positive patients treated with ARC-520 and only modest ALT elevations during or following treatment suggest minimal loss of hepatocytes. The implications for chronic HBV are significant because many hepatocytes in the parenchyma have integrated HBV. When they divide the clonal population with HBV integrants expands. Over time, this could result in maintained HBsAg expressed from integrated HBV, even if HBV DNA production is falling, which occurs frequently as patients age. If hepatocytes with integrated HBV are gradually replaced by oval cells and infection of these cells is inhibited by NUC therapy and/or other therapeutics that prevent reinfection, such as Hepcludex (previously Myrcludex-B), for example, the net result would be loss of HBV DNA. This would apply to cccDNA as well as integrated HBV. Indeed, two recent reports demonstrated that long-term NUC treatment results in loss of HBV DNA, both cccDNA and integrated HBV [51,52]. Although the percentages of patients able to reach functional cure through NUCs or PEG-IFN treatment are low, this process typically takes years [43]. The length of time may be due to the importance of slow replacement of hepatocytes. If high percentages of HBV-infected hepatocytes are killed by an immune-mediated mechanism, however, there could be increased risk of hepatocytes with integrated HBV expanding to replenish the liver. This effect could appear as increased HBsAg expression. The extent of hepatocyte killing, therefore, may prove critically important for new modalities of treatment.

RNAi can serve as a cornerstone for treatment combinations because it not only reduces virus but also the viral proteins that suppress the immune response. Viral proteins hide the viral nucleic acids with their pathogen-associated molecular patterns (PAMPs) from the innate immune system. Limited ALT elevations during and following RNAi treatment even while HBV parameters decrease bode well for a potentially gentle approach to functional cure with minimal liver damage. Preliminary evidence suggests RNAi treatment may improve prospects for younger patients with high amounts of cccDNA. Further studies are needed to evaluate what combinations of therapeutics, and perhaps even timing or sequencing of therapy, can result in high percentages of functional cure.

## Data Availability

All data relative to the study are available in the referenced publications.
